# The Law Relating to Lunatics and Persons of Unsound Mind.—II

**Published:** 1903-02-21

**Authors:** J. E. R. Stephens

**Affiliations:** Barrister-at-Law, Author of "Digest of Public Health Cases"


### HOSPITAL ADMINISTRATION.

### CONSTRUCTION AND ECONOMICS.

### THE LAW RELATING TO LUNATICS AND PERSONS OF UNSOUND MIND.—II.

By J. E. R. STEPHENS, Barrister-at-Law, Author of "Digest of Public Health Cases."

THE rules in *McNaghten's case*, given in the preceding article, have been most adversely criticised. It has been pointed out that the House of Lords had no legal right to ask, and that the judges were under no legal obligation to answer, the questions, since the constitutional right of the House of Lords to take the opinion of the judges arises only on appeals or when the House is acting in its legislative capacity. Again, it has been argued that the rules afford no guidance as to how cases of what is called *moral insanity*, in which the character of a person is suddenly changed for the worse or is by nature insanely perverted, should be dealt with; and, lastly, attention has been called, in connection with the doctrine of partial insanity, to the incongruity of supposing that a man who is admittedly subject to an insane delusion can reason sanely upon that delusion, and calmly argue with himself as to the extent to which he ought to allow it to influence his actions. This last criticism may furnish good ground for an application of the doctrine of modified responsibility in the class of cases with which it deals. But it must be pointed out that in thus localising the range of the immunity from punishment which insane delusion confers, the criminal law is merely following the course which, *mutatis mutandis*, the civil law has now with general acceptance, both in the legal and medical profession, adopted in questions of capacity, and which is moreover sanctioned by the disciplinary system that prevails in all institutions for the insane. It is chiefly in connection with cases of "moral insanity"—a generic term covering all those cases in which the moral faculties are more obviously deranged than the mental (kleptomanias, erotic manias, pyromanias, homicidal manias, irresistible impulses, etc.), that the rules in *McNaghten's case* have proved unsatisfactory in their practical application. But in recent years a gloss has been put in some cases upon the words "know" and "nature and quality," which has prevented any appreciable injustice from being done to accused persons by the apparent limitation of the rules to cases of ordinary derangement of the intellectual faculties. The gloss is that no man who is deprived by mental disease (*semble*, whether affecting his reason or his will) of the power of passing a rational judgment on the moral character of the act he is about to do, *knows its nature and quality* within the meaning of the rules referred to. The trend of decisions in the American Courts is adverse to this criterion of responsibility laid down in *McNaghten's case.*

Down to 1883, where a plea of insanity at the time of the commission of the offence was successfully set up, the jury returned a verdict of "not guilty on the ground of insanity." But the Trial of Lunatics Act, 1883 (46 and 47 Vict. c. 38) substituted for this a verdict of "guilty but insane," the idea of the Legislature being that the formal conviction recorded in the new verdict might have a deterrent effect on the mentally unstable.

Whether the prisoner was sane or insane at the time the act was committed is a question of fact triable by the jury, and dependent upon the previous and contemporaneous acts of the party. Evidence of insanity of ancestors or blood relations is admissible. So is evidence of illness exhausting the brain. Medical evidence is not essential. Mere absence of any evidence of motive for a crime is not a sufficient ground upon which to infer mania. Upon a question of insanity, a witness of medical skill may be asked whether, assuming certain facts, proved by other witnesses, to be true, they, in his opinion, indicate insanity, but not whether, having heard the whole evidence, he is of opinion that the prisoner at the time he committed the alleged act was of unsound mind. But a medical witness, who never saw the prisoner previously to the trial, but who was present during the whole trial and the examination of the witnesses, cannot, in strictness, be asked his opinion as to the state of the prisoner's mind at the time of the commission of the alleged crime, or his opinion whether the prisoner was conscious, at the time of doing the act, that he was acting contrary to law, or whether he was labouring under any and what delusion at the time, because each of these questions involves the determination of the truth of the matter deposed to, which it is for the jury to decide. Nor can such a witness, although present in court during the whole trial, be asked whether, from the evidence he has heard, he is of opinion that the prisoner at the time he did the act was insane, nor whether the act with which the prisoner is charged was in his opinion an act of insanity, for these are the very points to be decided by the jury. Counsel will not be allowed, upon a question of insanity, to quote in his address to the jury the opinions of medical writers as expressed in their books.

Drunkenness, which produces a perfect though temporary frenzy or insanity, usually denominated *dementia affectata*, or acquired madness, was formerly held not to excuse the commission of any crime, and an offender under the influence of intoxication could derive no privilege from a madness voluntarily contracted, and was regarded as equally answerable to the law as if he had been in the full possession of his faculties at the time. But legal opinion has altered on this subject, and it has been ruled that upon an indictment for murder the intoxication of the defendant may be taken into consideration as a circumstance to show that the act was not premeditated. The same has been held in cases o[illegible] attempts to commit suicide. When the crime alleged [illegible] such that the intention of the accused is one of its constitu[illegible] elements, the jury may look at the fact that he was in dr[illegible] in considering whether he formed the intent necessary to c[illegible]stitute the crime. And a man who, while under the influe[illegible] of delusions caused by *delirium tremens*, killed another [illegible] the belief that he was a wild animal, has been held [illegible] criminally responsible.

By the Inebriates Act, 1898 (61 and 62 Vict. c. 60) it [illegible] enacted, by s. 1 subs. 1, that "where a person is convicted [illegible] indictment of an offence punishable with imprisonment [illegible] penal servitude (*i.e.* of non-capital felony and most mi[illegible]demeanours), if the court is satisfied from the evidence th[illegible] the offence was committed under the influence of drink, [illegible] that drunkenness was a contributing cause of the offence, a[illegible] the offender admits that he is, or is found by the jury to b[illegible] a habitual drunkard, the court may, in addition to, or in substitution for any other sentence, order that he be detained in any State inebriate reformatory, or in any certified inebriate reformatory, the managers of which are willing to receive him." The term habitual drunkard is defined by s. 3 of the Inebriates Act, 1879, as meaning "a person who, not being amenable to any jurisdiction in lunacy, is notwithstanding, by reason of habitual intemperate drinking of intoxicating liquor, at times dangerous to himself or herself, or to others, or incapable of managing himself or herself, and his or her affairs." The definition appears to apply only to the habitual use of alcohol, and not to include the use of narcotics. The Act of 1898 does not appear to accept the view that drunkenness falling short of insanity is a defence on a charge of crime, but gives the courts latitude in treatment of crimes directly or indirectly due to drunkenness by permitting the reformative sentence as an alternative to the ordinary punishment for a crime. The doctrine of criminal responsibility in cases of drunkenness due to alcohol is equally applicable to mental or bodily conditions caused by the drinking of narcotics, or non-alcoholic stimulants, or exciting drugs, or their hypodermic injection.

A correct appreciation of the nice distinctions which English law makes between different degrees of insanity ought ever to be borne in mind by medical practitioners when they are called upon to give evidence in a court of law. For this reason it will be well to point out in detail the questions which English law in each case propounds as a test whether a patient is legally "insane" or not. Such a question may arise with reference to the alleged insane person's (*a*) treatment, (*b*) civil rights, (*c*) criminal liability, or (*d*) capacity either to be a witness or to make or revoke a will. As to treatment, there are three separate tests; the application of each varies according to what is the exact legal form in which the general question is raised.

(*a*) On a question whether a person is so insane as to render him a subject proper for treatment as insane—

1. If it be desired to have him found lunatic by inquisition, the legal question is, whether he is so unsound of mind as to be incapable of managing himself and his affairs.

2. If it be desired to place him in an asylum, the legal question for determination is, whether he is a proper person to be taken charge of and detained under care and treatment.

3. On an indictment against a person for a violation of the Lunacy Laws, by illegally receiving a lunatic, the question simply is whether the person so received was or was not "insane," or of unsound mind, in the sense in which the word is medically and scientifically used.

(*b*) On a question as to the civil rights of an alleged insane person—

4. In all civil cases to which an alleged insane person is a party, other than actions by such a person for what is called false imprisonment, and actions against him on a contract, the question whether such person is insane or not has no legal importance whatever, and need not be discussed, being, in point of law, wholly immaterial. This is equally the case whether the action be one by the alleged insane person, or one against him.

5. If a person has taken upon himself to restrain an alleged insane person without complying with the formalities prescribed by the Statute Law, and the person so restrained afterwards brings an action against him for what is in law called "false imprisonment," the person who has so acted can only justify what he has done by showing that the plaintiff was dangerous to himself or others: and accordingly in any such action for "false imprisonment," the question will be whether the alleged "lunatic" was so insane as to be dangerous to himself or others.

(6) In an action against an insane person, or an alleged contract made by him, the legal questions are: first, whether such person was, as a fact, so insane as to be unfit to make a contract of the kind in question; and next, whether his insanity was known to the other contracting party.

(*c*) On a question in a Criminal Court as to the criminal liability of an alleged insane accused.

7. The legal question is: whether, at the time when the act in question is alleged to have been committed, the accused "knew right from wrong," with regard to such act.

(*d*) On the question as to the capacity of an alleged insane person to make or revoke a will.

8. The legal question is: whether the testator was at the time of the alleged testamentary act, suffering from such insanity as may reasonably be supposed to have influenced (or, in one view of the law, which did actually influence) such testamentary act.

The eight different questions which may thus be raised as to whether a person is legally insane, in other words is "insane" so far as regards the particular act under consideration, must not be confused with the division into the two great classes—idiots and lunatics—which is in theory made by English law. That on a question of alleged insanity there should be no less than eight different tests possible, with regard to any one of which a witness may be called upon to testify, or, in the case of a medical or other skilled witness, to express an opinion, is a fact, the practical importance of which cannot be over-estimated.

(*To be continued.*)

